# Arthroscopic isolated long head of biceps tenotomy in patients with degenerative rotator cuff tears: mid-term clinical results and prognostic factors

**DOI:** 10.1007/s00590-020-02787-z

**Published:** 2020-09-10

**Authors:** Egbert J. D. Veen, Ashvin V. Boeddha, Ronald L. Diercks, Ydo V. Kleinlugtenbelt, Ellie B. M. Landman, Cornelis T. Koorevaar

**Affiliations:** 1 Department of Orthopedic Surgery and Traumatology, Deventer Hospital, P.O. Box 5001, 7400 GC Deventer, Nico Bolkesteinlaan 75, The Netherlands; 2 Department of Orthopedic Surgery, University Medical Center Groningen, University of Groningen, Groningen, The Netherlands

**Keywords:** Long head of biceps tendon, Biceps, Tenotomy, Rotator cuff tears, Degenerative, Arthroscopy

## Abstract

**Introduction:**

The long head of biceps tendon is frequently involved in degenerative rotator cuff tears. Therefore, this study explored the clinical results of an isolated biceps tenotomy and identified prognostic factors for improvement in pain and function.

**Materials and methods:**

Between 2008 and 2017, an arthroscopic isolated biceps tenotomy was performed on 64 patients with a degenerative rotator cuff tear (> 65 years). Primary outcome was patient-perceived improvement in pain and function. Potential prognostic factors for improvement in pain and function were identified.

**Results:**

A perceived improvement in pain was reported in 78% of the patients at three months after surgery and in 75% at a mean follow-up of 4.2 years (1–7 years; *n* = 55). A perceived improvement in function was observed in 49% of patients at three months and in 76% of patients at follow-up. Patients with a preoperatively normal acromiohumeral distance (> 10 mm) reported an improvement in pain and function significantly more often. Retraction of the supraspinatus tendon Patte 3 was significantly associated with worse functional outcome.

**Conclusions:**

A biceps tenotomy can be a reliable treatment option for patients with symptomatic degenerative cuff tears who fail conservative treatment and have a normal acromiohumeral distance (> 10 mm).

## Introduction

Degenerative rotator cuff tears are common in the aging population, and most tears are asymptomatic. Some patients may develop symptoms, but conservative treatment is still effective in most cases [1–3]. When conservative treatment fails, operative treatment can be challenging. Although rotator cuff repair is an effective procedure in the younger population, age is associated with less satisfactory results and a higher rate of re-tears [4, 5]. French orthopedic surgeons popularized arthroscopic tenotomy of the long head of biceps tendon (LHB) as a treatment option for patients with degenerative rotator cuff tears [6]. A tenotomy of the long head of biceps is found to produce an earlier pain relief compared to tenodesis [7] and minimal residual symptoms [8]. Also biceps surgery in combination with a rotator cuff repair, superior outcome is seen [9]. Both Walch et al. and Boileau et al. reported satisfactory results after this procedure in patients with degenerative rotator cuff tears who were not willing to participate in a long rehabilitation period after a rotator cuff repair or an irreparable tear [10, 11]. In all these studies the LHB tenotomy is often performed in combination with other procedures, less is known about the effect of an isolated LHB tenotomy. The optimal treatment for individual patients with a rotator cuff tear is still unclear in terms of which patients do well after conservative treatment, which patients benefit from isolated LHB tenotomy and which patients should be preferably treated with a rotator cuff repair. Recent publications show good clinical results in selected cases of rotator cuff repairs in elderly patients [12–14]. Other operative and more invasive treatment options for degenerative cuff tears are procedures like reversed shoulder arthroplasty (RSA), superior capsular reconstruction, lower trapezius tendon transfer and latissimus dorsi muscle transfer. The advantages of an arthroscopic LHB tenotomy are the short operation time, low risks for complications and a limited rehabilitation period compared to the other operative treatment options.

The aim of this study was to explore the clinical results of isolated LHB tenotomy in patients with degenerative rotator cuff tears and to identify potential prognostic factors.

## Materials and methods

### Study design

This is a retrospective longitudinal cohort study.

### Participants

Patients with a degenerative rotator cuff tear treated with an arthroscopic LHB tenotomy between 2008 and 2017 were included. Minimal follow-up was 12 months. Indications for an arthroscopic LHB tenotomy in these patients were a clinically and radiologically confirmed symptomatic degenerative rotator cuff tear, and age above 65 years, and failure of conservative treatment (of at least 6 months) including physiotherapy and cortisone injections, or patients with a symptomatic irreparable rotator cuff tear and failure of conservative treatment (of at least 6 months). Patients with a pseudoparalysis were excluded. Rotator cuff repair was not considered standard treatment for patients above age of 65 years with degenerative rotator cuff tears in our clinic during the study period. Other reconstructive options were considered not suitable, for this, often retired, patient population. If a cuff arthropathy was seen, a RSA was performed. Surgery was performed in a general teaching hospital by two dedicated shoulder surgeons (CK, YK). Patients were excluded when glenohumeral osteoarthritis was observed on plain radiographs, in the case of symptomatic osteoarthritis of the acromioclavicular joint, and if a fracture/dislocation of the operated shoulder occurred during the follow-up period. Approval of the Medical Ethical Committee was obtained (no: 180446), and all patients gave written informed consent. The STROBE guidelines were followed [15].

### Surgical technique

Arthroscopic surgery was performed in beach-chair position under an interscalene block of the brachial plexus. A posterior portal was used to enter the glenohumeral joint, and routine diagnostic arthroscopic evaluation was performed. An anterior working portal was used for instrumentation. Specific details of the rotator cuff and LHB were recorded for each patient. LHBT tenotomy was performed by sectioning the tendon at the origin at the superior labrum with an ablation device. We noted that in hypertrophic LHBs the tendon did not retract out of the glenohumeral joint after sectioning. In those cases, the intra-articular portion of the tendon was resected. No additional surgery was performed in any of the cases. Postoperatively patients wore a collar and cuff for two days, and passive range-of-motion exercises were started immediately. Active mobility exercises were started two days after surgery, as tolerated.

### Outcome measures

Primary outcome was patient-perceived improvement in pain and function after shoulder surgery, measured with two anchor questions in which patients were asked to indicate on a global rating scale how much their pain (pain anchor) or function (functional anchor) had changed after surgery [16, 17]. The response options for the anchor questions were: completely recovered (7), much improved (6), slightly improved (5), unchanged (4), slightly worse (3), much worse (2) and worse than ever (1). Secondary outcomes were the disabilities of the arm, Shoulder and Hand (DASH) score, VAS pain scores, patient satisfaction, and the EuroQol-5D (EQ-5D). The DASH score is a 30-item, self-report questionnaire designed to measure physical function and symptoms in people with any musculoskeletal disorder of the upper limb [18]. The DASH has been shown to be reliable, valid and responsive in patients with shoulder disability [19] and has been validated in Dutch for patients with a disorder of the upper limb [20]. Pain intensity was measured with the VAS pain score, where a score of 0 points represents no pain and a score of ten points represents unbearable pain. Patients were given a visual scale and were asked to point to the best representation of their pain. VAS pain at rest and VAS pain during activity were obtained. Patient satisfaction was scored by asking the patients whether they were very satisfied, satisfied, somewhat satisfied, disappointed or unhappy with the result of surgery. The EQ-5D is a quality-of-life questionnaire with five questions about mobility, self-care, usual activities, pain/discomfort and anxiety/depression. Five potential prognostic factors were designated: fatty infiltration of the infraspinatus muscle [21], retraction of the supraspinatus tendon, [22] type of LHB lesion, anterosuperior cuff tear [23] and acromiohumeral distance [24]. According to the recommendation of Peduzzi, we selected one candidate prognostic factor for every ten cases [25].

### Data collection

Two weeks prior to elective shoulder surgery, orthopedic patients were seen at an outpatient clinic by an independent physiotherapist. The following preoperative demographic and clinical variables were prospectively collected: age, gender, dominant shoulder and duration of shoulder complaints. The DASH score and the VAS pain score at rest and during activity were scored. After surgery, the following data were recorded: classification of type of LHB lesion [26] and whether the right or left shoulder was operated on. Three months after surgery, improvement in pain and function was scored with anchor questions for all included patients and recorded in the medical reports.

The patients received a print questionnaire at follow-up containing anchor questions about improvement in pain and function, DASH score, VAS pain at rest and during activity, patient satisfaction and the EQ-5D. All follow-up data were collected independently by the research unit of our orthopedic department using standardized case report forms and a study-specific database. If patients were re-operated, they filled in a questionnaire just before the second surgery, including anchor questions about improvement in pain and function, DASH score and VAS pain at rest and during activity. All second surgeries were considered the endpoint of the follow-up for these patients and were subsequently included in the analysis.

A standard measurement was performed of the acromiohumeral interval on an anteroposterior radiograph with the arm in neutral position. In this study, a modified classification of the acromiohumeral distance according to Saupe et al. [24] was used, considering more than 10 mm a normal acromiohumeral distance.

Retraction of the supraspinatus tendon was scored on an MRI scan using the Patte classification [27], and fatty infiltration of the infraspinatus muscle was scored using the Fuchs classification [28]. If no MRI scan was present before surgery, supraspinatus tendon retraction was scored during arthroscopy.

### Statistical analysis

Patient characteristics are presented as frequency counts and percentages for categorical variables and as mean and standard deviation (SD) for continuous variables. Differences between categorical variables were tested using the Chi-square test and dependent samples t test for continuous variables. IBM SPSS 24 was used for statistical analyses, and *p* values of < 0.05 were considered significant.

Five potential prognostic factors were investigated: retraction of the supraspinatus tendon, fatty infiltration of the infraspinatus tendon, presence of LHB lesion, anterosuperior cuff tear, acromiohumeral interval. Potential prognostic factors were first explored using cross tables and Chi-square tests. For comprehensibility and clinical applicability, categorical variables were computed into dichotomous variables. Variables that had significant associations with outcome measures were tested in multiple regression analyses.

## Results

### Description of the study population

**Fig. 1:**
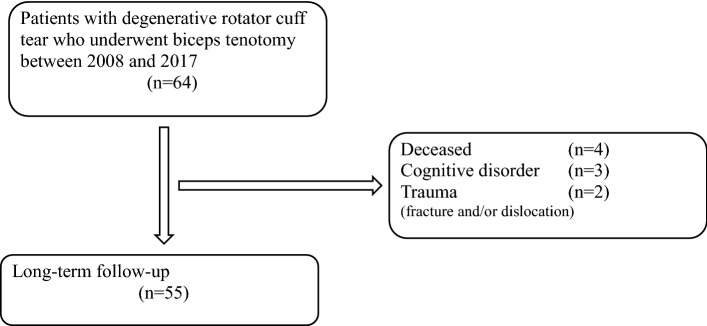
Inclusion flowchart

The study period included 64 patients. Nine patients were excluded from follow-up: one patient had a traumatic shoulder dislocation with a permanent lesion of the axillary nerve three years after surgery, and one patient sustained a fracture of the proximal humerus one year after surgery. Four patients died before follow-up, and three patients were not able to fill in a postoperative questionnaire due to a cognitive disorder. Figure 1 shows a flow diagram with study enrolment and follow-up.

Of the 55 patients included in the long-term follow-up, mean age was 72.1 years (SD 5.9) at the time of surgery. Demographic, clinical and radiological data at baseline are presented in Table 1. On clinical examination before surgery, no patient in the study population had a pseudoparalysis. An MRI scan was made before surgery in 31 out of 55 patients (56%) and an ultrasound in eight patients (15%). An isolated supraspinatus tear was present in 33 patients (60.0%), a posterosuperior cuff tear in 12 patients (21.8%), an anterosuperior cuff tear in eight patients (18.2%) and a three tendon tear in two patients (3.63%). Retraction of the supraspinatus tendon Patte stage 2 was seen in 15 patients and Patte stage 3 in 22 patients. Fatty infiltration of the infraspinatus muscle grades ‘severe’ was observed in 21% of patients. Table 2 presents the different LHB tendon lesions observed in the study population. A hypertrophy of the LHB was present in 44% of patients. The presence of a hypertrophic LHB was not associated with retraction of the supraspinatus tendon, two or three ruptured cuff tendons, fatty infiltration of the infraspinatus muscle or acromiohumeral distance.

**Table 1:** Patient characteristics (*n* = 55)

Age (years), mean (SD)	72.1 (5.9)
Male	21 (38.2%)
Right shoulder	31 (56.4%)
Dominant side	36 (65.5%)
Duration of symptoms (months), mean (SD)	19.9 (25.4%)
Isolated SSP tear	33 (60.0%)
SSP + ISP tears (posterosuperior cuff tear)	12 (21.8%)
SSP + subscap tears (anterosuperior cuff tear)	8 (18.2%)
SSP + ISP + subscap tears	2 (3.63%)
Supraspinatus tendon retraction (Patte)	
Unknown	12 (21.8%)
Grade 1	6 (10.9%)
Grade 2	15 (27.3%)
Grade 3	22 (40.0%)
Infraspinatus muscle fatty infiltration (Fuchs)	
Normal	7 (12.7%)
Moderate	16 (29.1%)
Severe	6 (10.9%)
Acromiohumeral distance	
≤ 10 mm	36 (65.4%)
> 10 mm	19 (34.5%)

*n* (%) unless otherwise noted, *SD* standard deviation, *SSP* supraspinatus, *ISP* infraspinatus, *Subscap* subscapularis

**Table 2:** Classification of long head of biceps tendon lesions (*n* = 55)

Type	Number	Percentage
Type 1: normal	19	(35%)
Type 2: tendinitis	1	(2%)
Type 3: SLAP lesion	0	(0%)
Type 4: partial tendon rupture	3	(6%)
Type 5: total tendon rupture	0	(0%)
Type 6: hypertrophy	24	(44%)
Type 7: subluxation	1	(2%)
Type 8: luxation	6	(11%)
Unknown	1	(2%)

### Primary outcome

Mean follow-up of these 55 patients was 4.2 years (range 1 to 7 years, SD 2.1). The clinical outcomes of this study are depicted in Table 3. An improvement in pain was reported in 78% of patients three months after surgery and in 75% at follow-up. In one patient, the shoulder deteriorated after a good initial clinical result, with a progression of the cuff tear into a massive cuff tear. An improvement in function was observed in 49% of patients three months after surgery and in 76% of patients at follow-up. Eight patients (15%) were re-operated because of unsatisfactory results after LHB tenotomy: six patients received a reversed shoulder prosthesis and one patient a hemi-shoulder prosthesis, and one patient underwent a latissimus dorsi transfer. No complications were recorded after surgery; one patient developed postoperative stiffness which resolved at follow-up.

**Table 3:** Clinical outcome scores

	Preoperative	Short-term follow-up *n* = 55 (3 months post-op)	Long-term follow-up *n* = 55 (mean 4.2 years)	*p* value
Anchor question pain, *n* (%)				
Completely recovered (7)		13 (23.6%)	15 (27.3%)	
Much improved (6)		23 (41.8%)	23 (41.8%)	
Slightly improved (5)		7 (12.7%)	3 (5.5%)	
Unchanged (4)		12 (21.8%)	10 (18.2%)	
Slightly worse (3)		–	1 (1.8%)	
Much worse (2)		–	3 (5.5%)	
Worse than ever (1)		–	–	
Anchor question function, *n* (%)				
Completely recovered (7)		3 (5.5%)	13 (23.6%)	
Much improved (6)		18 (32.7%)	22 (40.0%)	
Slightly improved (5)		6 (10.9%)	7 (12.7%)	
Unchanged (4)		24 (43.6%)	9 (16.4%)	
Slightly worse (3)		3 (5.5%)	1 (1.8%)	
Much worse (2)		1 (1.8%)	3 (5.5%)	
Worse than ever (1)		–	–	
DASH score (*n* = 30), mean (SD)	46.1 (17.1)		26.5 (22.8)	*p* < 0.001
VAS pain activity (*n* = 40), mean (SD)	8.2 (1.3)		3.0 (3.0)	*p* < 0.001
VAS pain rest (*n* = 40), mean (SD)	2.6 (1.9)		1.7 (2.3)	*p* = 0.045
Patient satisfaction, n (%)				
Very satisfied			20 (36.4%)	
Satisfied			16 (29.1%)	
Somewhat satisfied			8 (14.5%)	
Disappointed			6 (10.9%)	
Unhappy			5 (9.1%)	
EQ-5D total score, mean (SD)			0.792 (0.157)	

*SD* standard deviation

### Secondary outcome

Both the preoperative and the long-term follow-up DASH scores were available for 30 patients. A significant improvement in DASH score from 46.1 (SD 17.1) preoperative to 26.5 (SD 22.8) postoperative was observed after an arthroscopic LHB tenotomy (*p* < 0.001). Preoperative and postoperative VAS pain scores were available for 40 patients. Pain during activity improved from VAS 8.2 (SD 1.3) preoperative to VAS 3.0 (SD 3.0) at follow up; 65% of patients were satisfied or very satisfied with the result of surgery, with 15% somewhat satisfied. The EQ-5D score was 0.792 (0.157).

### Prognostic factors

Exploratory analysis showed that patients with an acromiohumeral distance of more than 10 mm reported significantly more often an improvement on the anchor questions for both pain (OR: 7.5, 95% CI: 1.5–37.4) and function (OR: 3.8, 95% CI: 1.1–13.6) at long-term follow-up (*p* = 0.014 and p = 0.042). For pain, there was major improvement or complete recovery in 90% of patients with a normal acromiohumeral distance (> 10 mm) and in 59% of patients with an acromiohumeral distance ≤ 10 mm.

Retraction of the supraspinatus tendon Patte grade 1/2 (versus grade 3) was significantly associated with functional improvement (OR: 7.2, 95% CI: 1.6–31.7). Hence, retraction of the supraspinatus tendon negatively influences outcome. No associations between presence of LHB lesion, fatty infiltration of the infraspinatus or anterosuperior cuff tear and anchor questions on pain and function were found. Groups were too small to provide sufficient power for multiple logistic regression analysis. All analyzed factors are depicted in Table 4.

**Table 4:** Prognostic factors and clinical outcome

	Anchor pain ≥ 6			Anchor function ≥ 6		
	OR	95% CI	*P* value	OR	95% CI	*P* value
Supraspinatus retraction (Patte 1/2)			NS	7.2	(1.6–31.7)	*p* = 0.009
ISP fatty infiltration (Fuchs 3)			NS			NS
Biceps tendon lesion			NS			NS
AH distance (> 10 mm)	7.5	(1.5–37.4)	*p* = 0.014	3.8	(1.1–13.6)	*p* = 0.042
Anterosuperior cuff tear			NS			NS

*NS* not significant, *CI* confidence interval, *OR* odds ratio, *AH* acromiohumeral

Anchor score 6: much improved

Anchor score 7: completely recovered

## Discussion

Our study shows good short- and mid-term pain relief was observed after arthroscopic LHB tenotomy in most of the elderly patients with degenerative rotator cuff tears and a failed conservative treatment with improvement on both functional and pain specific questionnaires. Acromiohumeral distance and retraction of the supraspinatus tendon were identified as prognostic factors for pain and function at mid-term follow-up. This is the first study assessing the clinical results after isolated LHB tenotomy in patients with a degenerative cuff rupture. This procedure can be a viable option for patients who are not candidate or do not have access to reconstructive surgery such as rotator cuff repair, superior capsular reconstruction or RSA.

Walch et al. reported satisfactory results in 87% of 307 patients with degenerative rotator cuff tears after arthroscopic LHB tenotomy at 4.7 years follow-up [10]. In that study, several patients had additional surgery: 36% an acromioplasty and 3% a distal clavicle resection. Boileau et al. reported 78% satisfactory results after 3.5 years follow-up after tenotomy or tenodesis of the LHB in patients with irreparable rotator cuff tears [11]. Patient satisfaction was comparable to the present study: 65% of patients were very satisfied or satisfied, and 15% of patients were somewhat satisfied.

The role of the LHB in shoulder pain has been debated for a long time. Boileau et al. described entrapment of the LHB in patients with degenerative cuff ruptures [29]. They noticed a deformation of the intra-articular portion of the LHB during arthroscopy and called this the hourglass biceps. The hourglass theory suggests that the hypertrophic intra-articular portion of the LHB leads to entrapment within the joint on elevation of the arm. The intra-articular portion of the tendon buckles and becomes incarcerated within the joint, inhibiting passive and active elevation and causing pain. Hypertrophy of the intra-articular tendon leads to a disproportion between the tendon and the cross-sectional size of the bicipital groove, preventing sliding of the tendon into the groove and leading to its entrapment. Leffert et al. [30] believe that hypertrophy represents a mechanism of functional compensation in the absence of a rotator cuff. Kido et al. [31] suggest the depressor theory and discuss the function of the LHB as a humeral head depressor, as well as its possible hypertrophy in cases of rotator cuff rupture. Our study promotes the idea that the intra-articular part of the LHB tendon plays role in the pain of patients with a symptomatic rotator cuff tear.

Acromiohumeral distance appeared to be of great importance in treating patients with symptomatic degenerative rotator cuff tears. Patients in the current study with a normal acromiohumeral distance (> 10 mm) reported significantly better improvement in pain and function after LHB tenotomy than patients with a shorter acromiohumeral distance (≤ 10 mm), irrespective of type of LHB lesion. Saupe et al. [24] studied acromiohumeral distance in patients with rotator cuff tears and used three groups: group 1 with a normal acromiohumeral distance (> 10 mm), group 2 with an acromiohumeral distance between 7 and 10 mm and group 3 with an acromiohumeral distance ≤ 7 mm. They observed that acromiohumeral distance correlated with size of the rotator cuff tear and degree of fatty degeneration of the infraspinatus muscle. Walch et al. observed superior results after LHB tenotomy in patients with an acromiohumeral distance greater than 6 mm and an association of fatty infiltration of the rotator cuff musculature with inferior results after biceps tenotomy [10]. In our study, the group of patients with an acromiohumeral distance of 7 mm or less was too small to make any comparison. Based on the studies mentioned above, it was decided to compare between more and less than 10 mm.

Limitations were the retrospective study design and the absence of a control group. The pain relief observed after LHB tenotomy could be the result of the tenotomy but might also be attributable to a surgical placebo effect, to the postoperative treatment of the physiotherapist or to a benign natural course. Patients included in this study had symptoms for a long period (mean duration of 19.9 months), and most patients were already being extensively treated by a physiotherapist before surgery. Interestingly, the pain relief after LHB tenotomy was already observed within three months postsurgery and was maintained at longer follow-up. This supports the concept of the LHB as a pain generator in patients with a degenerative cuff tear. Furthermore, the improvement in 19.6 points on the DASH score exceeds the minimal important clinical difference (MICD) of 10.8 points [32]. In this study, we used anchor questions as specific questions about pain and functional improvement after surgery. Patients were asked in a single question to indicate how much their function or pain had changed since baseline. The anchor questions may be affected by recall bias [33]. Tashjian and colleagues classified these anchor questions as retrospective assessment of outcome improvement [34]. They compared prospective and retrospective assessment of functional outcome after rotator cuff repair. Retrospective assessment had fair correlations with prospectively determined improvement, for example with the DASH score. Patient satisfaction was more highly correlated with retrospective evaluations than with prospective improvement in functional outcome measures. Moreover, we have to consider possible confounding biases of the prognostic factors. The acromiohumeral distance will be more narrowed in patients with a much retracted supraspinatus and fatty infiltration of the infraspinatus. However, groups were too small to provide sufficient power for multiple logistic regression analysis.

## Conclusion

Arthroscopic long head of biceps tenotomy in patients with degenerative cuff tears and failed conservative treatment can be a reliable treatment option when the acromiohumeral distance is normal (> 10 mm), with both good short- and mid-term pain relief and functional outcomes. Other treatment options should be considered when the acromiohumeral distance is shorter in patients with degenerative cuff tears.
